# Feldenkrais ‘Functional Integration’ Increases Body Contact Surface in the Supine Position: A Randomized-Controlled Experimental Study

**DOI:** 10.3389/fpsyg.2018.02023

**Published:** 2018-10-24

**Authors:** Matthias Brummer, Harald Walach, Stefan Schmidt

**Affiliations:** ^1^Pro Corpore, Center for Physical Therapy and Feldenkrais Method, Fürth, Germany; ^2^Department of Pediatric Gastroenterology, Poznan Medical University, Poznan, Poland; ^3^Department of Psychology, University Witten-Herdecke, Witten, Germany; ^4^Change Health Science Institute, Berlin, Germany; ^5^Department of Psychosomatic Medicine, Medical Faculty, Medical Center – University Hospital Freiburg, Freiburg, Germany; ^6^Institute for Transcultural Health Studies, European University Viadrina, Frankfurt, Germany

**Keywords:** Feldenkrais, functional integration, body image, pressure measurement, randomized cross-over trial

## Abstract

Feldenkrais ‘Functional Integration’ (FI) is a widely used type of body work with a focus on the continuous integration of body sensations and awareness with movement. The method is, amongst others, known for improving balance in aging populations, but also for its ability to relax muscles. With participants treated in the supine position FI is potentially changing the surface area of the body in contact with the surface on which a participant is lying. So far, no prior study has assessed this claim. We evaluated objectively and subjectively if a treatment with FI would induce changes in pressure and contact surface of the body on the mat. Thirty volunteers received an individual treatment with FI, in a randomized order on both sides of the body. Pressure and contact surface was documented with the Xsensor-Measurement-System. Subjective sensations were assessed with a self-report scale. Due to two parallel assessments alpha-level was adjusted to α = 0.025. We found that pressure and contact surface of the body on the mat significantly changed after the treatment (factor time: *p* < 0.0001, $\eta_{p}^{2}$ = 0.90). We also found that pressure and contact surface increased significantly on the left side for the group that started with the left side first (time × group *p* = 0.016; $\eta_{p}^{2}$ = 0.62), but less so on the right side for the group that started with the right side first (time × group: *p* = 0.056) although there was still a substantial effect size ($\eta_{p}^{2}$ = 0.54). The subjective reports confirmed the physical measurements. In conclusion our results demonstrate for the first time that the treatment with the Feldenkrais method changes muscle tone leading to a more relaxed supine position with respect to pressure and contact surface on the mat.

## Introduction

The Feldenkrais-method is named after its founder, the Israeli physicist and engineer Moshe Feldenkrais (1904–1984), and is described as a method of motion-related, somatic learning (Buchanan, 2010). This method is widely applied in medical, athletic, pedagogic, and artistic fields (Russell, 2004). The Feldenkrais-method is generally used, and accepted in certain health systems, for instance in Germany, for special indications such as postural deformity, limitations of the musculoskeletal system, dorsal pain, neurological diseases, chronic pain, developmental disorders of children and adolescents, psychosomatic and stress-related illnesses (Hufelandgesellschaft, 2009, p. 33). The Feldenkrais-method is used worldwide and extensively in the United States, Australia, and Germany (Malmgren-Olsson et al., 2001).

There are two reviews assessing the effectiveness of the Feldenkreis-method (Ernst and Canter, 2005; Hillier and Worley, 2015). The more recent one reports 20 RCTs of which 13 showed a superiority of the Feldenkrais intervention compared to a control condition. Study populations and outcome measures were highly heterogeneous since Feldenkrais is a somewhat generic approach. The risk of bias was medium to high. The authors performed meta-analyses for subsets of studies regarding balance training in aging populations and found positive effects with clinical meaningful effect sizes (Hillier and Worley, 2015). More recent studies complement this evidence by demonstrating significant results in physical functioning for individuals with intellectual disability compared to wait-list (Torres-Unda et al., 2017) and by demonstrating significant improvement in quality of life and depression in a sample of Parkinson patients compared to a control group receiving only educational lessons (Araújo et al., 2015). Another RCT could not find significant differences for patients with chronic low back pain when comparing Feldenkrais to Back School (Paolucci et al., 2017). Within the Feldenkrais-method two options for application are described. Exercise in groups is called ‘Awareness through Movement’ (ATM), where clients perform the movement through verbal instruction on their own. Individual treatment and exercise, which is the subject of this study, is called ‘Functional Integration’ (FI). This treatment is carried out predominantly through directed movements in a non-verbal way, i.e., the patient’s body is passively and gently mobilized and moved by the hands of the Feldenkrais-Practitioner. For instance, the practitioner may softly lift an arm from the mat and perform a range of motions with the patient, who just lets this happen and observes the effect. The movements can be quite small or sometimes rather large and playful (Bearman and Shafarman, 1999). From a historical perspective, FI was developed as the first application of the Feldenkrais-method (Feldenkrais, 2013, p. 109).

During and after a treatment with the Feldenkrais-method FI, patients very often describe a change of bodily perceptions. Patients quite frequently state that the contact of their body with the mat has changed through treatment. Mostly they state, if for example only one side of the body has been treated, that this side has a larger contact surface with the mat. Patients also frequently describe that the treated side now feels bigger in size, surface, and more voluminous. This change of self-perception could be related to a factual change of muscle tension as a result of the intervention described above and lead to a physically enlarged contact surface with the mat. Feldenkrais proposes as a mechanism for this effect of FI: ‘the tonicity gets more even and lower’ (Feldenkrais, 1987, p. 209). However, it is also possible that the contact-surface is not in fact changing, but only the subjective perception of contact. This would mean a change of self-awareness without a physical change of the contact surface with the mat. Hence, it is necessary to know whether those reported changes in perception are actually due to physical changes in surface contact, or purely subjective.

In the study conducted here the contact of the body with the mat was examined, which is perceived through the afferent system of proprioceptors (Joraschky et al., 2006). In the context of the Feldenkrais-method, Dunn’s work has shown that sensory changes can be generated through Feldenkrais-exercises. There, Feldenkrais-exercises were performed imaginatively with one side of the body and sensory changes were gathered in a self-devised questionnaire. Nine out of eleven participants stated a significant change in bodily perception (*p* < 0.04), both sensory and motoric (Dunn and Rogers, 2000).

In the RCT on chronic lower back pain comparing Feldenkrais to back school (Paolucci et al., 2017) interoceptive awareness was assessed as well with the Multidimensional Assessment of Interoceptive Awareness Questionnaire (MAIA) (Mehling et al., 2012). Patients in both groups showed highly significant improvements in interoceptive awareness with the Feldenkrais group being slightly better than the Back School group (*p* = 0.056). Of related interest is a neuroscientific study assessing neural activity in relation to Feldenkrais stimulation (Verrel et al., 2015). Participants in this study were stimulated at the feet while being in a MRI scanner. They showed, amongst others, a higher resting state activity in the regions of interest after the application of the Feldenkrais techniques.

Although it is difficult to exclude response bias in such subjective data, they formed the basis for the assumption that through the Feldenkrais-method a change of internal and external perception of the body might be generated. A randomized controlled trial analyzed, if it is possible to enhance the ability of kinaesthetic discrimination in the shoulder-arm-area by Feldenkrais-exercises. This three-armed study showed that the group doing Feldenkrais-exercises displayed a significant improvement (*p* < 0.01) of its ability to discriminate compared to the control group (Czetczok, 1987, p. 133).

Thus, there are only scarce scientific data answering the question, whether FI actually does change elements of the body experience, and if so, all the data available are subjective. Usually, instrumental-objective and verbal-subjective methods are recommended for the examination of body schema and body percept (Joraschky et al., 2006). So far, there is no study assessing objective indicators in relation to reported interoceptive changes. In our study the objective was to find out whether the subjectively reported enhanced contact surface described by patients receiving FI is due to a change in the participant’s self-perception only, or due to a physical change of the body’s contact surface with the mat that can be measured objectively. More specifically, we hypothesized that (1) the contact surface and pressure will increase after treatment with FI and (2) that this increase is area specific and can be only found in the body side treated (right or left). Therefore, in this experimental study healthy volunteers that had signed up for FI session received a treatment with FI, with a random choice of which side of the body to start with. This served as our independent variable. Pressure and contact-surface on the mat were measured objectively and participants were asked about their subjective experience. Treatment was performed by a qualified and licensed Feldenkrais-Practitioner and licensed physiotherapist (Matthias Brummer).

## Materials and Methods

### Participants

**FIGURE 1:**
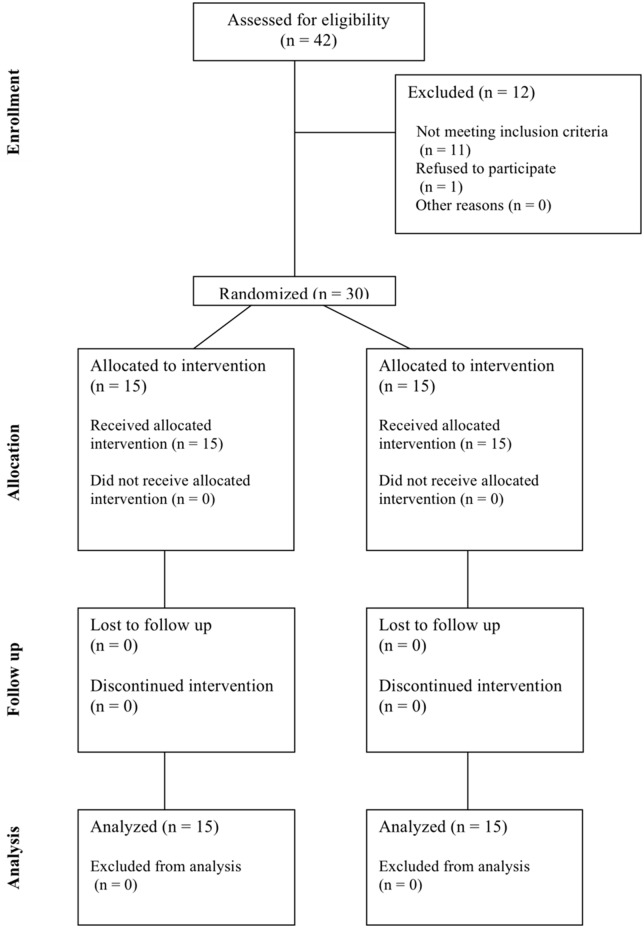
CONSORT flow chart.

Thirty healthy participants (see Figure 1), mean age 37.9 years (*SD* = 14.17, range: 20–65), were recruited from routine physiotherapy-clinic and gave written informed consent to participate. Nineteen of the participants were female, and 11 were male. Special care was taken that all patients with symptoms, diseases or medications that could influence their self-awareness were excluded. All participants were informed thoroughly about the procedure and the implementation of the study and gave their written consent. They received a free FI treatment for providing their data. All data were treated confidentially according to data protection laws. The study follows the guidelines of Good Clinical Practice and meets the recommendations of the Helsinki Declaration World Medical Association (2008). All participants were to receive a FI treatment anyway and no other change to the FI procedure was introduced except the contact-surface measurement and a questionnaire.

Therefore, the study was not deemed ethically questionable and no special ethical clearance was sought.

### The Xsensor-Measurement-System

The Xsensor-Measurement-System is a pressure mapping system which leads to exact and reproducible results when measuring pressure in industry or medicine, and is applied as a standard industry development system (Cork, 2007).

In the medical field, the Xsensor-technology is mostly used for the development of wheelchairs and mattresses to prevent bedsores. Among others, different chair cushions for paralyzed wheelchair-patients were examined to facilitate an ideal pressure distribution (Trewartha and Stiller, 2011). Mattresses to prevent bedsores were also compared with each other using this system (Hardin et al., 2000); and the pressure distribution of mattresses in hospitals was analyzed in the context of different body types (Moysidis et al., 2011). Finally, the system was applied to compare four different surgical tables during surgical interventions of 80 patients to evaluate pressure distribution (Keller et al., 2006). Thus, the system used here has been already widely in use and proven it’s utility. Kim et al. (2012) give an overview of research in this field.

**FIGURE 2:**
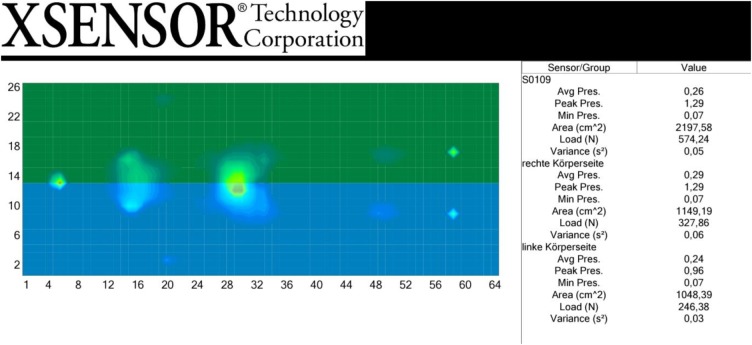
Example for Xsensor-measurement frame.

In this study the Xsensor-Measurement-System was used to measure the body pressure and the contact surface on the mat during an individual Feldenkrais-treatment ‘FI.’ Here, the participant lies on his back on a sensor mattress (Xsensor PX100:26.64.01, Xsensor Technology Corporation) which is connected to a computer. The sensor surface is 81.2 by 203.2 cm in size, and fitted with 1664 measurement points. This equals a sensor density of 31.75 mm. The data processing is achieved by X3 MEDICAL software v6.0. The complete treatment is video-recorded in real time. The Xsensor-Measurement-System determines the pressure and the contact surface of the participants. The data are visualized in a 2D-false-color-display, with colors corresponding to pressure zones (see Figure 2: Example for Xsensor-measurement frame) and transformed into numeric values in the statistic module of the software. For each measurement point T0, T1, and T2 we used one single measurement frame after breaks of five breaths.

### Subjective Measurement

For measuring subjective sensations a validated German instrument, the KEKS-scale, was employed. The KEKS is a means for registering current bodily perceptions, i.e., the body-percept (Joraschky et al., 2006, p. 106). The test contains 20 items including two distractor items that measure hypersensitivity (marker item: ‘I can feel my cerebellum’). The internal consistency of the complete test, without distractor items was α = 0.93. The coefficient of reliability for the two distractor items was α = 0.71. Thus, the scale has good reliability, as well as convergent and discriminant validity. KEKS showed a significant positive correlation with dispositional self-awareness (Filipp and Freudenberg, 1989) of *r* = 0.27 (*p* < 0.05). To verify the discriminant validity, the KEKS-data of adults exercising Hatha-Yoga were compared with adults that did not exercise Hatha-Yoga. Four factors could be completely replicated, which speaks in favor of constructive validity. Altogether 65% of the variance of these four factors could be explained (Joraschky et al., 2006).

The KEKS-scale was adapted for the purposes of this study, i.e., we doubled the 12 items of the scale that referred to body parts that can be felt on either side into left and right sided perceptions. This is in effect a doubling of the body-relevant items of the scale. We included the hypersensitive items only as a cross-check, and none of our participants was hypersensitive, indicating that we could use the data.

### Pre-investigation and Recruitment of Participants

We carried out pre-investigations using the Xsensor-Measurement-System. Measurements of five persons served to identify the measurement range and to calibrate the measurement system. This fine calibration is necessary to gather reliable data. Data were collected similar to the setting planned for the study at three points in time: before the treatment (T0), after treatment of one side of the body (T1), and after treatment of the other side of the body (T2) when the treatment was finished. All participants that called in the physiotherapy-clinic for an FI treatment were informed about the option to participate in this study. They received a session for free for allowing measurements, and all subsequent patients consenting to participate were included in the study.

### Randomization

Randomization was conducted using the program iRandomizer Numbers (Shmoopi, 2013). It was decided by a random number generator which side would be treated first, with all even numbers denoting the right side of the body to start with.

### Procedures, Data Collection, and Data Management

Participants lay on their back on the treatment mat. They were asked to put their arms along the side of the body and to stretch out their legs. This was the starting position at every point of measurement. After a pause of five breaths measurement T0 started. The recording of the body contact (pressure and surface) followed, using the Xsensor-Measurement-System. Subsequently, the randomly chosen side of the body was treated for 25 min. After finishing the intervention at one side of the body, and after another break of five breaths, measurement T1 followed. Next, the other side of the body was treated for another 25 min. After this treatment was finished, and after another break of five breaths, the final measurement T2 was performed, and the treatment was finished. The participants were asked to answer the KEKS-items verbally and the answers were documented, so as to not change their posture, immediately after the Xsensor System had recorded the measurements. The Xsensor-System recorded all measurement data automatically in a file, in combination with a real-time video recording. Thus, three one-point-measurements for the points of measurement T0, T1, and T2 were conducted after breaks of five breaths.

### Statistical Analysis

For the statistical evaluation, one single measurement frame was used for each point of measurement T0, T1, and T2. The values of the maximal pressure, minimal pressure, average pressure, contact surface, load (contact surface × pressure) and variance (of an initial entirety of data of one sensor for a single frame; the average pressure on the sensor is displayed) are calculated for every frame. The value of the minimal pressure can be regarded as a cross-check value here. To eliminate interferences such as hair, the sensor is calibrated such that the measurement starts only at 0.07 N/cm^2^. The measurement unit for pressure is N/cm^2^ and for surface centimeter squared.

For a differentiated consideration of the results, analyses of the whole body at the three points of measurement were performed as well as a division of the whole frame into different sensor groups along the body axis, i.e., divided into right side and left side.

This was done to differentiate between the changes of the right and left side of the body, the right and left side of the chest and the right and left side of the pelvis. As a result, an evaluation of the whole body, the right and left side of the body, the right, and left side of the chest as well as the right and left side of the pelvis are available for each of the three points of measurement, together with the six values described above. Xsensor data were imported into Statistica (version 8.0) and processed further.

With respect to power considerations, we had no prior effects size estimations since this is the first study of this type. Thus, we could only make a rough estimation with a conservative approach by defining at least a medium size effect-size and arrived at *N* = 30 (1-β = 0.8). Descriptive methods and analyses of variance with repeated measurements, both for the right and left side of the body were used for evaluation. Since this is a first and hence exploratory study, we steered a middle ground between inflation of *p*-values through multiple testing and too restricted modeling by adopting the following strategy. We investigated the data visually for potential effects, following the theoretical rationale of Feldenkrais FI which predicts that the side treated first should display stronger effects. Since the variables are correlated we chose highest pressure, area and load and included these into a multivariate repeated measurement analysis of variance (MANOVA) with the group sequencing as a between factor and the three measurement points as within factor. We expected differential effects between sides and/or groups that should show as interaction effects, as well as effects over time, but no between group effects, which would rather be an indicator for carry-over or other experimental artifacts. Hence we focused mainly on the measurements according to differential sides of the body. We deliberately decided against merging the data of the right and left body side into one analysis. Such a procedure would have obliged us to code the respective body side according to the randomization into ‘side treated first’ and ‘side treated second.’ This would imply to ignore differences between the right and the left side of the body and to treat them as interchangeable. As a result of this approach we arrived at separate analyses for the right and the left body side. This made a correction of the alpha-level necessary in order to control for multiple analyses. Thus, alpha was set to *p* = 0.025. We report $\eta_{p}^{2}$ as effect size, which indicates the variance explained by the respective variable without the variance explained by other predictors. As a rule of thumb, values of 0.01 are considered as small of 0.06 as medium, and 0.14 as large effect sizes, respectively (Lakens, 2013). Analytical strategies were decided in advance in a study protocol.

## Results

Thirty participants between 20 and 65 years of age were invited to this study. Nineteen of the participants were female, and 11 were male. Their average age was 37.9 years (*SD* = 14.2). Such a gender distribution of 63% females and 37% males is fairly typical for patients and studies using the Feldenkrais-method (Busch, 2011). Seventy three percent of the participants had already had experiences with methods for enhancing bodily perception, or with relaxation techniques, such as autogenic training, yoga, the Feldenkrais-method, concentrative movement therapy or Thai Chi. All participants were free of diseases that would influence bodily perception, nor did they take any drugs.

A full multivariate model of all variables covering the right and the left side of the body was calculated. Results are displayed in Tables 1, 2.

**Table 1:** Full multivariate model of RM ANOVA with all variables covering the right side of the body.

Effect	Wilk’s lambda	*F*	*df* effect/error	*p*-value	$\eta_{p}^{2}$
Intercept	0.0007	6543.53	5/24	<0.0001	0.999
Group	0.8965	0.55	5/24	0.73	0.103
Time	0.104	16.34	10/19	<0.0001	0.896
Interaction Group^∗^Time	0.452	2.30	10/19	0.056	0.548

**Table 2:** Full multivariate model of RM ANOVA with all variables covering the left side of the body.

Effect	Wilk’s lambda	*F*	*df* effect/error	*p*-value	$\eta_{p}^{2}$
Intercept	0.0004	9684.40	5/24	<0.0001	0.999
Group	0.8749	0.69	5/24	0.64	0.125
Time	0.093	18.62	10/19	<0.0001	0.907
Interaction Group^∗^Time	0.380	3.10	10/19	0.016	0.619

To explore potential interactions further, we calculated specific models for areas of the body. The shoulder area showed no differential effects. However, the pelvis region showed clear effects of time and interaction effects. The results of a multivariate model using only the variables ‘highest pressure,’ ‘area,’ and ‘load’ both for the left and the right side of the pelvis are presented in Tables 3, 4.

**Table 3:** Multivariate model of RM ANOVA with the variables ‘highest pressure,’ ‘area,’ and ‘load’ for the right side of the pelvis region.

Effect	Wilk’s lambda	*F*	*df* effect/error	*p*-value	$\eta_{p}^{2}$
Intercept	0.0171	497.60	3/26	<0.0001	0.982
Group	0.9251	0.70	3/26	0.55	0.074
Time	0.328	7.84	6/23	0.0001	0.671
Interaction Group^∗^Time	0.407	5.58	6/23	0.001	0.592

**Table 4:** Multivariate model of RM ANOVA with the variables ‘highest pressure,’ ‘area,’ and ‘load’ for the left side of the pelvis region.

Effect	Wilk’s lambda	*F*	*df* effect/error	*p*-value	$\eta_{p}^{2}$
Intercept	0.0190	446.90	3/26	<0.0001	0.980
Group	0.9596	0.36	3/26	0.77	0.040
Time	0.194	15.88	6/23	<0.0001	0.805
Interaction Group^∗^Time	0.414	5.41	6/23	0.001	0.585

**FIGURE 3:**
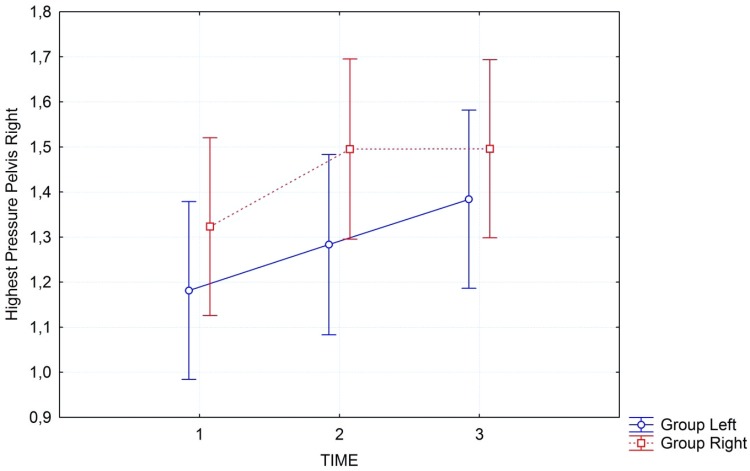
Interaction graph of “highest pressure” in the pelvis region on the right side of the body, for the two groups separately: group left (blue line) started with left side; group right (red line) started with right side. Error bars refer to 95% confidence intervals.

**FIGURE 4:**
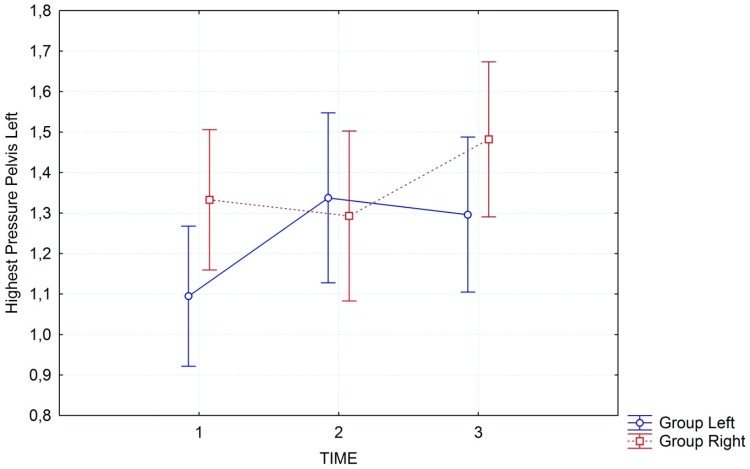
Interaction graph of “highest pressure” in the pelvis region on the left side of the body, for the two groups separately: group left (blue line) started with left side; group right (red line) started with right side. Error bars refer to 95% confidence intervals.

Sample interaction graphs for the variables ‘Highest Pressure’ in the pelvis region right and left are presented in Figures 3, 4.

This objectively measured change in pressure and area covered by the body was partially confirmed by subjective reports. The questionnaire measuring bodily sensations showed a clear and significant time trend in multivariate repeated measures ANOVA as well, with ‘felt area covered’ and ‘felt pressure’ as the variables, first for the right and then for the left hand side. While the right hand side showed both a clear time trend (Wilk’s lambda = 0.536; *F*_4/25_ = 5.40; *p* = 0.003; $\eta_{p}^{2}$ = 0.46) and a significant interaction between group and time (Wilk’s lambda = 0.546; *F*_4/25_ = 5.18; *p* = 0.003; $\eta_{p}^{2}$ = 0.45), the left hand side showed only a clear cut time trend (Wilk’s lambda = 0.583; *F*_4/25_ = 4.46; *p* = 0.007; $\eta_{p}^{2}$ = 0.42) but no significant interaction (Wilk’s lambda = 0.729; *F*_4/25_ = 2.31; *p* = 0.085; $\eta_{p}^{2}$ = 0.27).

## Discussion

To the best of our knowledge, this is the first study that demonstrated objectively measured changes occurring after Feldenkrais FI in healthy volunteers. Participants were healthy with an interest in FI. Our exploratory analysis shows that the gentle passive movements that are directed toward one side of the body first, are indeed having a differential effect. This is what participants in FI sessions report, but it was unclear until now, whether this subjective feeling can be objectively confirmed. In the global analysis we see that the interaction between group and time is significant for the left side of the body but fails to reach the adjusted significance level for the right side of the body... The respective effect sizes for both sides are quite substantial with a large amount of variance explained. Time effects are highly significant in all analyses, but this is what would be expected. A treatment like FI is actually demonstrating effects. The body is relaxing and thus is measured with higher pressure on a larger surface area covering the mat. The fact that interactions can be observed is most remarkable. These are stronger for the group that starts with the left side first, but still visible for the group that starts with the right side first. The effects become more pronounced when we focus on the pelvis, the region of the body that normally is felt most heavily and has a large contact-surface with the mat by default. As can be seen from the interaction graphs the pressure increases differentially on the side that is treated first. Although also the other side of the body shows a concomitant increase in pressure, this is stronger for the side treated first. In the pelvis region this can be seen very clearly in the significance of the interaction, while for the full body this effect is somewhat weaker, yet still observable.

The fact that this effect is easier to observe, if treatment starts with the left side is not easy to explain. One could speculate that this is related to the left side being the non-dominant side, and hence a treatment that is focusing on this side first will produce a stronger effect.

Our treatment shows a strong effect over time with very large effect sizes $\eta_{p}^{2}$, demonstrating that the gentle movements of FI in fact relax muscle groups and the body over time, resulting in stronger pressure and a larger contact surface area. It is reassuring to see that there is no group difference. This means that our randomisation procedure resulted in comparable groups, and carry-over effects are not an issue.

The objective data are partially reflected in subjective reports about sensed areas covered by the body and pressure felt. Also here we see a clear time trend on both sides, and a significant interaction between time and group, with the group treated on the right side first showing a clear effect in that they sensed their body on the right side more strongly and covering a larger area. This was only partially reflected in the group treated on the left side first. This discrepancy is maybe related to the fact of handedness with most people’s dominant side being the right hand side. It can be assumed that subjective availability of sensations is likely to be better for the dominant side. Thus, although the effect was objectively stronger for the group starting with the left side first, the subjective reports were clearer for the group starting with the right hand side first, and in general terms, the objective results were reflected in the subjective data. With respect to the comparison of objective and subjective reports of body contact and pressure, hardly any other research could be found. The only report identified by us came from the car industry. It confirmed positive correlations of subjective and physical characteristics in assessing seating comfort (Park et al., 1998). Regarding the improvement of interoception our data are in alignment with the results of a study by Paolucci et al. (2017) that documents improved interoceptive awareness after a Feldenkrais intervention in chronic lower back pain patients.

With respect to the objective assessment our study is, to our knowledge, the very first to perform such measurements of FI or any other body-work therapy, hence there is no point of comparison. The closest experimental study we could find is a study assessing resting state activity in the brain during FI stimulation (Verrel et al., 2015). Here a higher resting state activity was measured after FI treatment, but it remains subject to future research to assess the relationship between these variables from neuroscience with the physical and self-reported variables assessed in our study. Therefore, a next step would be to replicate our findings, potentially with more focused hypotheses. Since our effect sizes are fairly large and observed power for all our effects were close to 1 except for the non-significant interaction term for the right-hand side, a replication study would not need to be much larger. It might be worthwhile to reduce the number of variables, and, for instance to only record maximum pressure and load. It would also be interesting to compare FI treatment with a second control condition of participants lying supine on the mat without any treatment for the same time, or a self-directed relaxation exercise. An interesting extension of our findings would be to study patients who have functional disorders, such as chronic tension pain. Our data surely represent a rationale for employing and studying Feldenkrais FI more widely, also in clinical contexts. Other questions which we did not address and that might be worthwhile addressing are, whether other methods of relaxation and body work, such as progressive muscle relaxation, Yoga, or autogenic training have comparable effects. The effect sizes we observed in a single session of 50 min were sizeable. Our data give a scientific basis to FI work and demonstrate that this kind of work deserves a more detailed attention by the community.

We conclude from our study that Feldenkrais FI indeed relaxes the body. This is visible through a larger area covered by the body and higher pressure, objectively measured. This change in pressure and coverage is dependent on the work itself, as it occurs differentially in strict dependence on the side where the work starts. This is more clearly visible on the left-hand side of the body.

## Author Contributions

MB conducted the study and the treatment, collected the data, and wrote a first draft of the manuscript. SS supervised the protocol formulation and the design and took part in writing. HW wrote part of the manuscript, conducted the analysis, and supervised the protocol formulation.

## Conflict of Interest Statement

MB runs a private practice and offers Feldenkrais functional integration as part of his work. The remaining authors declare that the research was conducted in the absence of any commercial or financial relationships that could be construed as a potential conflict of interest.
